# Treatment of age-related visual impairment with a peptide acting on mitochondria

**DOI:** 10.1242/dmm.048256

**Published:** 2022-02-21

**Authors:** N. M. Alam, R. M. Douglas, G. T. Prusky

**Affiliations:** 1 Burke Neurological Institute, 785 Mamaroneck Avenue, White Plains, New York, NY 10605, USA; 2 University of British Columbia, Department of Ophthalmology and Visual Sciences, 2550 Willow Street, Vancouver, BC Canada V5Z 3N9; 3 Weill Cornell Medicine, Department of Physiology and Biophysics, 1300 York Avenue, New York, NY 10065, USA

**Keywords:** Aging, Behavior, Drug treatment, Mitochondrial therapeutic, Restore vision, Visual impairment, Optokinetic tracking

## Abstract

Age-related visual decline and disease due to neural dysfunction are major sources of disability that have resisted effective treatment. In light of evidence that visual impairment and mitochondrial dysfunction advance with age, we characterized age-related decline of spatial visual function in mice and investigated whether treatment of aged mice with the mitochondrion-penetrating peptide elamipretide that has been reported to improve mitochondrial function, would improve it. Impaired photopic acuity measured by using a virtual optokinetic system emerged near 18 months and declined to ∼40% below normal by 34 months. Daily application of the synthetic peptide elamipretide, which has high selectivity for mitochondrial membranes that contain cardiolipin and promotes efficient electron transfer, was able to mitigate visual decline from 18 months onwards. Daily application from 24 months onwards, i.e. when acuity had reduced by ∼16%, reversed visual decline and normalized function within 2 months. Recovered function persisted for at least 3 months after treatment was withdrawn and a single treatment at 24 months delayed subsequent visual decline. Elamipretide applied daily from 32 months onwards took longer to take effect, but substantial improvement was found within 2 months. The effects of age and elamipretide treatment on contrast sensitivity were similar to those on acuity, systemic and eye drop applications of elamipretide had comparable effects, scotopic spatial visual function was largely unaffected by age or treatment, and altered function was independent of variation in optical clarity. These data indicate that elamipretide treatment adaptively alters the aging visual system. They also provide a rationale to investigate whether mitochondrial dysfunction is a treatable pathophysiology of human visual aging and age-related visual disease.

## Abstract (precis)

**Summary:** Age-related decline in vision in mice is substantially prevented or restored in response to treatment with a peptide that comprises mitochondrial affinity and improves mitochondrial function.

## INTRODUCTION

Visual decline related to normal aging contributes to disability and reduces health span (Owsley, 2011, 2016). Hardening (presbyopia; Lafosse et al., 2020) and clouding (cataracts; Asbell et al., 2005) of the lens are common optical consequences of aging that can seriously impair visual function. The development of devices and procedures (e.g. corrective eyewear; lens replacement surgery, etc.) to reduce refractive error in these conditions has drastically reduced the burden of age-related optical visual impairment in the world, though it remains substantial (Holden et al., 2014). Independent of optical factors, untreatable decline of vision due to age-related neurological dysfunction is also a major source of impairment (Owsley, 2011). This can present as the deterioration of spatial visual function (Elliott, 1987), luminance modulation (Woi et al., 2016), binocular processing (Arani et al., 2019), color perception (Karwatsky et al., 2004), sensitivity to motion (Habak and Faubert, 2000) and dark adaptation (Gaffney et al., 2012; Jackson et al., 1999), among other impairments. However, some visual functions, such as blur adaptation, appear to remain intact with age (Elliott et al., 2007). Age also predisposes the visual system to develop age-related diseases, such as glaucoma, diabetic retinopathy (DR) and age-related macular degeneration (AMD). These blinding diseases are common – approximately one in three elderly persons has some form of vision-reducing eye disease by the age of 65 years (NEI, Eye Health Data and Statistics) – and have risen with medical advances that have extended lifespan. Unfortunately, neither the etiology of age-related visual decline nor the mechanisms of how age contributes to blinding diseases are sufficiently well understood to enable effective treatment. Thus, despite the prospect of increased longevity, the elderly face reduced quality of life with increased risk of disability from falls, immobility and depression linked to visual impairment (Court et al., 2014).

Since age itself contributes to untreatable visual decline, understanding the natural history and pathophysiology of visual aging has clinical relevance. However, the typically large variability between individuals regarding the effects of aging on human vision, and the often-small sample sizes in clinical studies are not optimal conditions for identifying fundamental pathophysiology of visual aging. Alternatively, using inbred mice in studies of visual aging, with their reduced subject-to-subject variability, has the potential to characterize the natural history of mammalian visual aging and help identify its neurophysiological substrates. Although there are reports of declining visual acuity with age in mice (Weber et al., 2015), a careful quantification of spatial visual function – acuity and contrast sensitivity – among the most widely used and clinically-relevant measures of visual function across the lifespan has, to our knowledge, not yet been completed.

Characterizing visual decline over the lifespan may also aid in understanding and modeling age-related retinal disease. Despite the link between age and disease, preclinical models of sporadic age-related blinding diseases have not routinely included advanced age (i.e. in mice older than 18 months) as a variable. Rather, they have focused on genetic mutations or physiological modifications that induce retinal degeneration in younger animals. Whereas this approach has deepened our mechanistic understanding of retinal degeneration, it has not necessarily advanced relevant rodent models of age-related blinding diseases – particularly for sporadic AMD, which is often modeled with mutations linked to inherited forms of disease, such as retinitis pigmentosa, e.g. The Royal College of Surgeons rat (Sauve et al., 2001; DiLoreto et al., 1998). The lack of age as a variable in preclinical models might have contributed to the problem that few interventions have been successfully translated for use in human age-related visual disease. Thus, understanding the contributions of age to visual decline, is likely to be an important step in determining what distinguishes age-related visual decline from age-related visual disease.

Numerous abnormalities in cellular physiology have been linked to aging (Takahashi et al., 2000; López-Otín et al., 2013), but among the most evident is that compromised mitochondrial bioenergetics is a regulator of age (Kauppila et al., 2017) and disease (Annesley and Fisher, 2019). Such dysfunction is measured as reduced ATP production, decreased membrane potential, oxidative stress, organelle swelling, cristae damage and decreased DNA copy number, among other deficiencies. Since mitochondria are present in all cells and mitochondrial function is required for a host of cellular functions – i.e. regulation of apoptosis, calcium buffering, nuclear genome signaling, production of reactive oxygen species (ROS), steroid synthesis, immune system signaling, regulation of cell cycle and cell growth, etc. (Bock and Tait, 2020; Tait and Green, 2012; Fang et al., 2016) – it is not surprising that the consequences of mitochondrial dysfunction are diverse, affecting a wide variety of physiological processes and organ functions. Consistent with this are reports that link dysfunctional mitochondria to aging (Bratic and Larsson, 2013) and a wide variety of diseases, including those that are age-related, i.e. cancer, metabolic disease and diabetes, inflammatory disease, neuropathy, nephropathy, cardiomyopathy, and also neurodegenerative conditions, i.e. Alzheimer's, Parkinson's and Huntington's disease (Swerdlow et al., 2017; Lin and Beal, 2006). In the visual system, mitochondrial mutation-linked dysfunction subserves Leber Hereditary Optic Neuropathy – the most common inherited mitochondrial disorder (Pilz et al., 2017). In addition, mitochondrial dysfunction, in the form of impaired mitochondrial dynamics – i.e. fusion and fission – has also been linked to primary open-angle glaucoma (Shim et al., 2016) and AMD (Nashine et al., 2019; Ferrington et al., 2016).

One option to test the hypothesis that mitochondrial dysfunction is a pathophysiology of age-related visual decline would be to treat aged mice (i.e. in mice, older than 18 months of age) with a pharmacological agent that targets mitochondria and improves mitochondrial function, followed by evaluating its ability to prevent or reverse visual decline. If the treatment were beneficial, it would provide evidence supporting mitochondrial dysfunction to be a pathophysiology of age-related visual decline and, by association, implicating mitochondrial dysfunction in age-related visual disease. In addition, a therapeutic target and a potential therapeutic agent to treat human age-related visual decline and disease, could be identified. Elamipretide (also known as SS31, SS-31, MTP-131) (Zhao et al., 2003, 2005; Schiller et al., 2000) is among the most promising candidate compounds to test this mitochondrial dysfunction hypothesis of visual aging. Elamipretide is a synthetic tetrapeptide with alternating aromatic-cationic repeats that are attracted to the negative surface charge of mitochondrial membranes enriched in cardiolipin (Berezowska et al., 2003), where it alters biophysical properties through electrostatic and hydrophobic interactions (Birk et al., 2013). Other work has linked effects of elamipretide treatment to the function of the electron transport chain (ETC) (Chavez et al., 2020; Allen et al., 2020) and to alterations of the biophysical properties of cardiolipin-enriched membranes (Mitchell et al., 2020). Cardiolipin plays a central role in the formation of mitochondrial cristae, the ETC, and in regulating the function of ETC complexes and ATP synthase (Mileykovskaya and Dowhan, 2014). Elamipretide also mitigates peroxidation of cardiolipin and inhibits cytochrome *c* peroxidase activity (Birk et al., 2013, 2014). The combined ROS reduction and apparent mitochondrial rejuvenating properties of elamipretide appear to benefit age-related decline in skeletal muscle (Siegel et al., 2013; Campbell et al., 2019), and kidney (Sweetwyne et al., 2017) and brain function (Tarantini et al., 2017). In age-related disease models, elamipretide has been effective at ameliorating dysfunction associated with hypertension (Dai et al., 2011), heart failure (Dai et al., 2013) and glaucoma (Wu et al., 2019). Elamipretide treatment has also shown to be effective at treating visual decline in a mouse model of diabetic retinopathy (Alam et al., 2015a), which has linked mitochondrial dysfunction to visual disease. Thus, in the study presented here, we tested the hypothesis that elamipretide prevents and reverses spatial visual decline in a mouse model of visual aging.

## RESULTS

### Spatial visual function declines with age

**Fig. 1.**
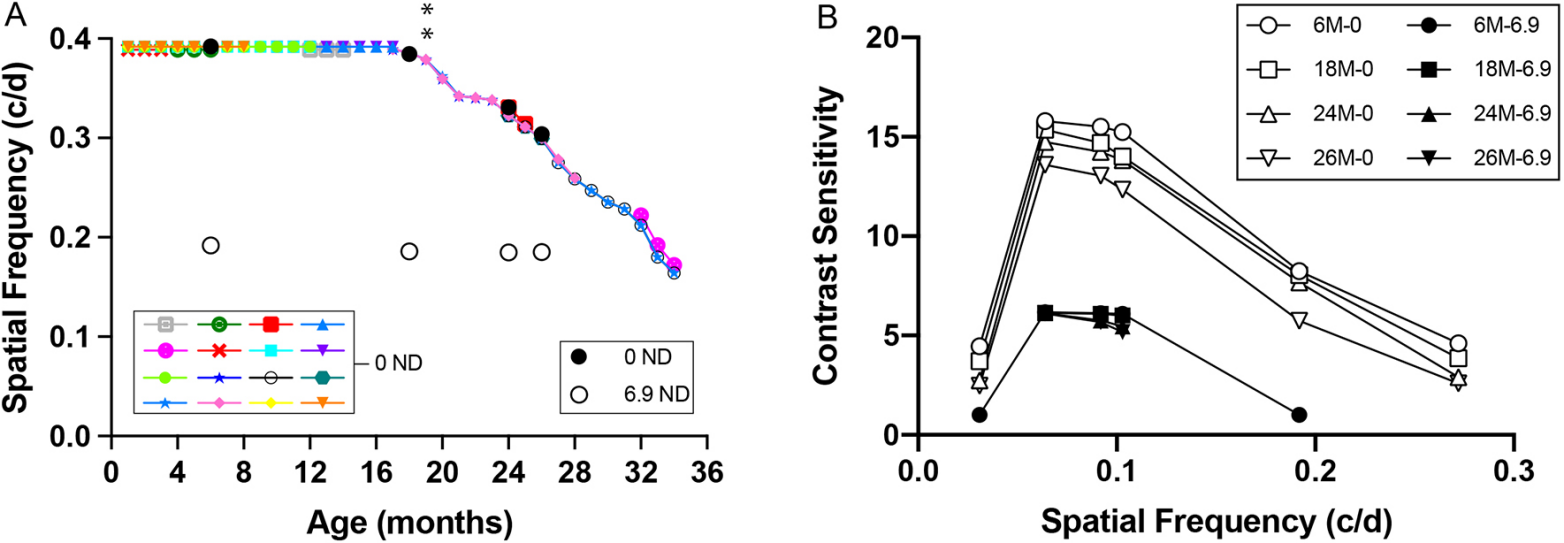
**Mouse spatial visual function declines with age.** (A) Spatial frequency thresholds. Colored symbols (inset on left; 0 ND) depict weekly average photopic measurements in 16 separate cohorts of mice (*n*=224). Function near 0.39 c/d was maintained in mice aged between 1 and 18 months, starting to decline thereafter (19 months onwards) comprising periods of increases and decreases (**P<0.01). Function at 34 months (0.16 c/d) was reduced by 59% relative to normal adult values. Black and white large circles (inset on right; 0 ND, 6.9 ND): One cohort (*n*=6) was measured under both photopic (0 ND, closed circles) and scotopic (6.9 ND, open circles) conditions. Photopic acuity substantially declined with age (6-26 months= −22.4%) in the group, scotopic acuity varied little over the same period (-2.8%). Bars indicate standard deviation (±s.d.) but are often smaller than the data symbols. (B) Contrast sensitivity function in mice aged 6, 18, 24 and 26 months, measured under photopic (0 ND) and scotopic (6.9 ND) conditions. Photopic function (open symbols) declined with age at all spatial frequencies. Scotopic function (closed symbols) did not change much with age at spatial frequencies near peak sensitivity. Symbols obstruct any ±s.d. values.

Acuity averaged to an ∼0.39 cycles per degree (c/d) from 1 month to 18 months of age (Fig. 1A). This value was consistent with previous reports of normal adult visual function using the same strain, apparatus and measurement procedures (Prusky et al., 2004, 2006; Douglas et al., 2005; Tschetter et al., 2013; Alam et al., 2015b). Function gradually declined after 18 months to ∼70% normal adult function at 34 months (0.39 c/d versus 0.12 c/d; *P*<0.01); the oldest cohort tested.

One group of mice was measured periodically when aged between 6 months and 26 months under both photopic and scotopic conditions, the results of which are also presented in Fig. 1A. Scotopic thresholds at 6 months averaged 0.19 c/d – characteristically lower than photopic thresholds (Prusky et al., 2004; Douglas et al., 2005; Alam et al., 2015b) – and varied little at 26 months (scotopic 0.192c/d to 0.187c/d, *n*=6, *P*<0.0001); photopic 0.39c/d to 0.30c/d, *n*=6, *P*<0.0001). This reveals that normal scotopic visual function was maintained over the same period in which photopic function was in decline. The trend of declining photopic visual function and more-stable scotopic visual function continued in measurements of contrast sensitivity (CS) in mice aged between 6 and 26 months, which is illustrated in Fig. 1B. The contrast sensitivity functions (CSFs) of 6-month-old mice were consistent with previous work using the same strain and measurement procedures (Prusky et al., 2004; Douglas et al., 2005; Alam et al., 2015b). Photopic function declined gradually across spatial frequencies in mice aged 6-24 months, and decline accelerated in those aged 24-26 months. Scotopic CS decreased little as a function of age. Only spatial frequencies near peak sensitivity were measurable by 18 months and, thus, a characteristic convex function could not be obtained. At maximum sensitivity, a modest decline was evident in mice aged 24 months compared with those aged 6 months.

### Elamipretide treatment can slow or reverse age-related visual decline

**Fig. 2.**
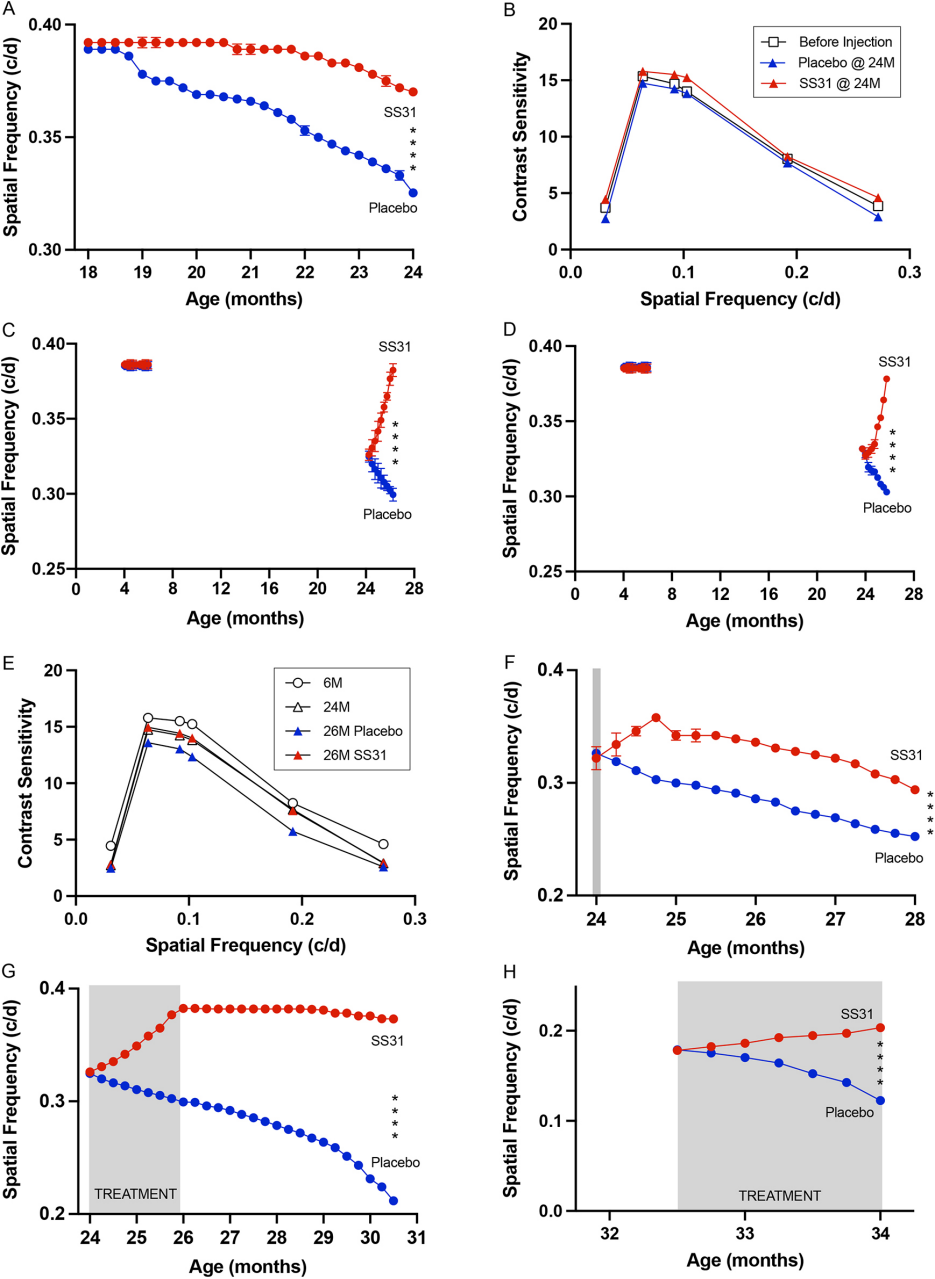
**Elamipretide at varying doses and administered by s.c. injection or eye drops delays and/or alleviates age-related photopic vision loss even in extremely old mice.** (A) Spatial frequency threshold. Mice treated with elamipretide by s.c. injection (SS31; red; *n*=6) from 18 months onwards maintained visual function within normal range for >2 months. Thereafter, the rate of decline was reduced compared to the placebo-treated group (blue; *n*=6; 5.5% versus 16.3% decline at 24 months). (B) Contrast sensitivity. Elamipretide treatment (s.c. injection; red triangles) from 18 months onwards led to slightly improved function relative to placebo-treated animals (blue triangles) and resulted in better function at most spatial frequencies compared with that of untreated animals before treatment was initiated (white squares). (C) Effect of daily s.c. treatment with elamipretide (SS31) on spatial frequency thresholds. Left side of plot: Spatial frequencies in drug-treated mice (0.39 c/d, *n*=10, red) from 4-6 months were similar to placebo-treated mice (0.39 c/d, *n*=10, blue). Right side of plot: At 24 months (*n*=52) decline reduced within 1 week and restored to normal function by 26 months (0.38 c/d) in drug-treated animals, whereas placebo-treated animals (*n*=49) continued to decline (0.30 c/d at 26 months). (D) Effect of daily elamipretide eye drop treatment on spatial frequency thresholds. Left side of plot: Drug-treated mice (red dots) at 4-6 months (*n*=10) were similar to those treated with placebo (*n*=10; 0.39 versus 0.39 c/d; blue dots). Right side of plot: The same treatment from 24 months (*n*=52) reversed decline within 4 weeks and restored normal function by 26 months (0.38 c/d), whereas placebo-treated animals (*n*=49) continued to decline (0.30 c/d at 26 months). (E) Effect of daily eye drop application of elamipretide on contrast sensitivity. Drug treatment (red triangles) maintained visual function at 24-month pre-treatment baseline values (open triangles) relative to placebo treatment (blue triangles) but did not restore function to 6-month pre-treatment baseline values (open circles). (F) Effect of single s.c. elamipretide treatment (red) at 24 months (gray shading) on photopic spatial frequency threshold (acuity; *n*=7). Function improved for 4 weeks after treatment and then paralleled age-related decline in placebo-treated mice (blue; *n*=7) until 28 months of age (0.28 c/d versus 0.25 c/d). (G) Effect of treatment withdrawal at 26 months. Mice were daily s.c. injected with elamipretide or placebo (gray shading) between 24 and 26 months. Treatment was stopped at 26 months. Near-adult normal function was maintained in drug-treated animals (red; *n*=8) for >4 months (29 months of age) after withdrawal, and much slower loss of function with age was exhibited thereafter relative to placebo controls (blue dots; *n*=8; 0.37 versus 0.21 c/d). (H) Treatment benefit of elamipretide on spatial visual impairment in advanced age. Daily elamipretide eye drop treatment (gray shading) from 32 months of age led to improved spatial frequency thresholds within 2 months (0.18 c/d versus 0.20 c/d) compared with loss of function in placebo-treated mice (0.18 c/d versus 0.12 c/d). Standard deviations (±s.d.) are shown as vertical bars throughout but are occluded by symbols in some panels; ****P<0.0001.

After characterizing a decline in photopic acuity and CS with age, we set out to determine whether elamipretide (referred to in figures as SS31) prevents the advancement of age-related visual impairment. For this, mice were treated daily from 18 months onwards with subcutaneous (s.c.) 0.9% saline (placebo) or elamipretide (SS31, 1 mg/kg). Photopic spatial frequency and contrast thresholds were measured regularly in the same animals until they were 24 months old. Fig. 2A shows that the elamipretide-treated cohort's photopic acuity was substantially preserved; the rate of decline was reduced in elamipretide-treated compared with placebo-treated mice (SS31=−0.0008097; Placebo=−0.002485). Indeed, normal photopic visual function was maintained for at least 10 weeks after treatment and, thereafter, the rate of photopic decline was slower compared with that in placebo-treated control mice. Compared to baseline measures at 18 months (0.39c/d), by 24 months (0.32c/d), photopic visual function in the elamipretide-treated cohort declined by 6% compared with 16% in the cohort treated with placebo. Fig. 2B shows a similar trend, i.e. elamipretide-preserving function with treatment from 18 months was evident in CS measurements, but the effect was not as straightforward to interpret. In general, elamipretide treatment led to a modest improvement of function; but, at some spatial frequencies, photopic visual function in treated animals was not different to that of controls. Together with the results of acuity measures, these data indicate that the treatment benefit of elamipretide on spatial vision was at the high spatial frequency range.

We then investigated whether photopic visual function could be restored in older, i.e. 24-month-old, mice – an age at which we observed degradation of 18% of photopic acuity and 5% of photopic CS at peak sensitivity (see Fig. 1). For this, 24-month-old mice were treated daily with s.c. injections of placebo or elamipretide, for a period of two months. Fig. 2C shows that acuity in elamipretide-treated mice improved relative to those treated with placebo within 1 week of initiating treatment (*P*<0.0001) and continued to improve thereafter. After 8 weeks of treatment, the threshold in the elamipretide-treated cohort returned to pre-decline values (*P*>0.9999) and was unchanged in young mice that had undergone the same course of treatment from 4 months of age showing that the application of elamipretide did not augment normal function.

To target the eye more directly and to explore the feasibility of using eye drops to deliver a therapeutic dose of elamipretide, we replicated the two-month course of s.c. injections with vehicle or elamipretide in 24-month-old mice by daily application of eye drops. Fig. 2D shows that, although eye drop application of elamipretide proved slower to improve function than s.c. application (5 weeks versus 1 week, respectively), eye drop treatment was able to restore photopic acuity to the same degree as s.c. injection over the course of 2 months. Fig. 2E shows that the CSF of aged mice after eye drop application of elamipretide for 2 months was maintained at 24-month pretreatment baseline values.

To investigate the potency and persistence of elamipretide to reverse age-related visual decline, different durations of treatment were employed, and acuity was measured in the same animals for 2-4.5 months after treatment. Fig. 2F shows data from 24-month-old mice treated with a single s.c. injection of placebo or elamipretide, followed by repeated measures of acuity for 4 months. This s.c. elamipretide treatment led to improved function within 1 week (*P*<0.0001), which continued to improve over the next 3 weeks. Thereafter, function declined at a rate that closely resembled the loss-of-function rate of the placebo-treated group until measurements were stopped at 28 months (SS31=−0.00395; placebo=−0.005218). In another experiment, mice were treated daily with s.c. injections of elamipretide or placebo when 24-26 months old (treatment that led to full recovery of function) (Fig. 2G). After treatment was suspended for 4.5 months (i.e. in mice being 30.5 months old), the elamipretide cohort maintained 97% of their recovered function, whereas that of the placebo group declined to 46% of normal adult function (Fig. 2G).

We also investigated whether elamipretide can improve visual function in extreme old age (i.e. aged 32 months or older), i.e. when vision is substantially more impaired. At 32 months, when ∼50% loss of visual function had occurred, mice were treated with daily s.c. injections of placebo or elamipretide for 2 months (Fig. 2H). Although there was little deviation in age-related visual decline in response to treatment with elamipretide for the first 2 weeks after treatment, function gradually improved thereafter. By the last measurement at 34 months, vision of the elamipretide cohort had improved significantly (*P*<0.0001), whereas that of the placebo group had declined to 31% of normal function (*P*<0.001; SS31 versus placebo treatment at 34 months *P*<0.001). This indicates that, once recovery commenced in the 32-month-old elamipretide-treated group, rate of improvement was slower than in animals that had begun the same 2-month treatment regimen at 24 months (Fig. 2, compare panels H and C).

**Fig. 3.**
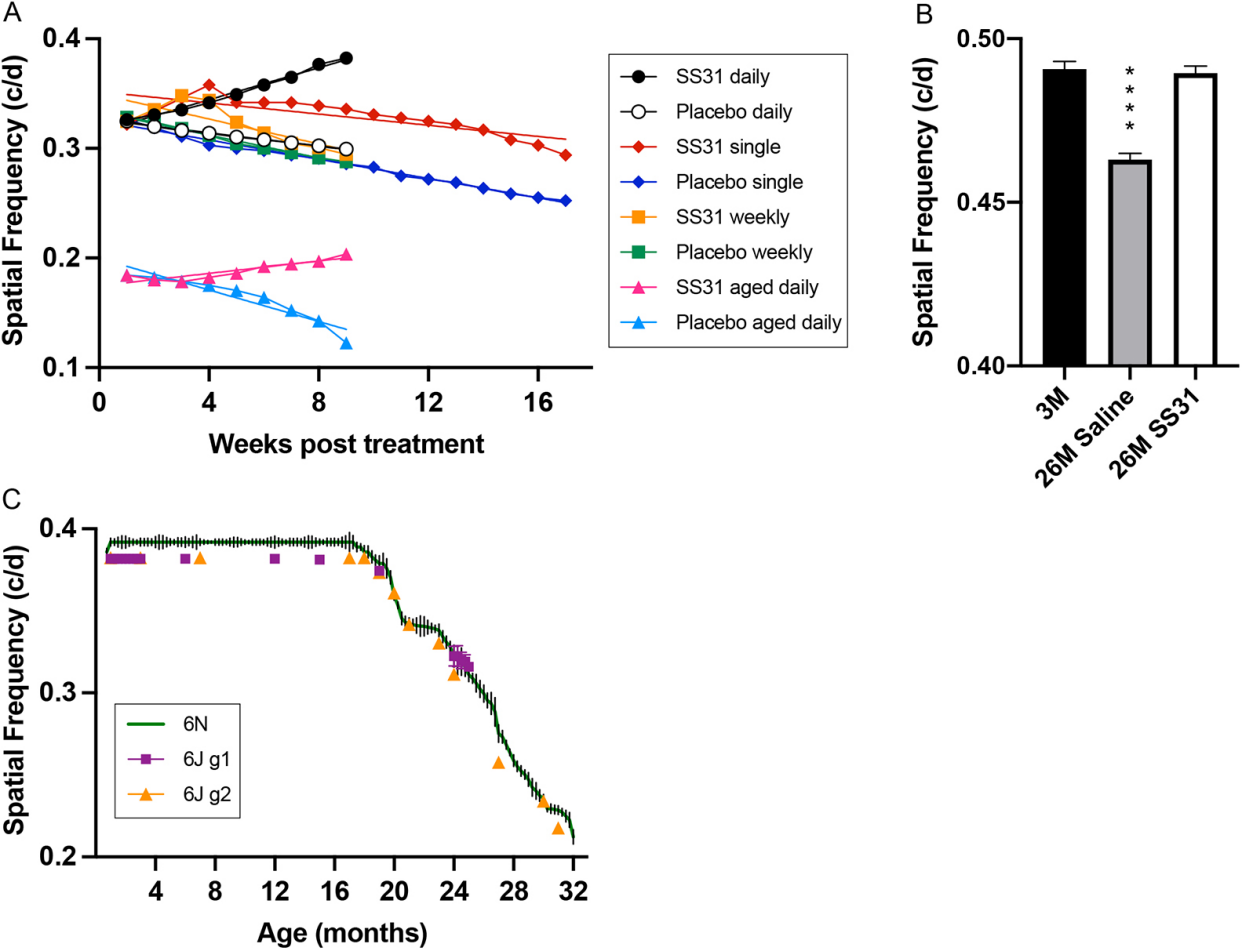
**Additional analyses and experiments.** (A) Comparison of elamipretide (SS31) treatment regimens. Spatial frequency threshold results shown in Fig. 2C, F, G and H plotted from start of treatment (see Key). Daily treatment from 24 months (black circles=SS31; white circles=placebo control) produced the largest benefit, the initial rate of improvement with treatment at 24 months appeared better when a single treatment (red diamonds=SS31; blue diamonds=placebo control) or weekly treatments (orange squares=SS31; green squares=placebo control) were used. The rate of recovery in response to daily treatment between 24 and 26 months (black circles) was faster than that in response to daily treatment from 32 months onwards (pink triangles=SS31; blue triangles=placebo control). Slope line is plotted for all plots. (B) Bar graph showing photopic temporal frequency thresholds of untreated mice at 3 months (3M, black), 26-month-old mice treated with saline (control) from 24-26 months (26M Saline, gray), and 26-month-old mice treated with elamipretide from 24-26 months (26M SS31, white). Bars indicate standard deviation (+s.d.). Results show temporal processing declines with age and can be restored to normal adult values by treatment with elamipretide. (C) Comparison of age-related decline in C57Bl/6N (plotted as vertical ±s.d. bars of data shown in Fig. 1A), and C57Bl/6J mice plotted as colored symbols for each cohort tested (Bars indicating ±s.d. are often smaller than and obstructed by symbols). Mature visual function (1-18 months) of C57Bl/6J mice was slightly reduced compared with C57Bl/6N mice, but the pattern of age-related visual decline was similar.

We used several elamipretide administration schedules to establish whether the peptide is effective at reversing age-related visual decline, and to gain insight into the kinetics of the drug's action. To enable direct comparisons between various treatment regimens, we plotted the results of experiments shown in Fig. 2C, F, G and H on the same time scale (Fig. 3A), allowing direct comparisons of rate and magnitude of elamipretide responses on acuity in response to treatment for 2 months or more. This revealed that, although daily treatment between 24 and 26 months (SS31 daily) had the greatest benefit, the initial rate of improvement in function was faster in response to a single treatment at 24 months of age or weekly treatments from 24 to 26 months (SS31 single or SS31 weekly, respectively). The rate of recovery in response to daily treatment at ages 24-26 months (SS31 daily) (slope=0.007310) was also faster than that in response to treatment at 32 months onwards (SS31 aged daily) (slope=0.002830) (Fig. 3A).

We also investigated whether temporal frequency thresholds were differed, depending at what age elamipretide treatment was provided. The threshold responses of vehicle-treated 26-month-old mice were lower than those of 3-month-old mice (*P*<0.001), and elamipretide treatment between 24 and 26 months was able to normalize the function (3M versus 26M SS31, *P*=0.6114) (Fig. 3B). This shows that temporal processing of photopic information, in addition to spatial processing, declines with age and can be improved by elamipretide treatment.

The core results in this study were generated using C57Bl/6N mice acquired from NIA colonies. C57Bl/6N mice have been reported to carry retinal degeneration 8 (rd8) mutation of the *Crb1* gene, which leads to an anatomical phenotype of retinal degeneration (Mattapallil et al., 2012). The C57Bl/6J strain, however, has been reported to carry a deletion of five exons within the nicotinamide nucleotide transhydrogenase (*Nnt*) gene, but does not appear to carry rd8 mutation (Ronchi et al., 2013). To determine whether age-related visual decline in C57Bl/6 mice varies according to sub-strain and/or retinal mutation, we compared the visual function between two C57Bl/6J mice cohorts (acquired from The Jackson Lab) raised in-house as they aged, with those generated in C57Bl/6N mice presented in Fig. 1A. Fig. 3C shows that, although the baseline adult function in C57Bl/6J mice was lower than that in the C57Bl/6N strain, the overall pattern of age-related visual decline was comparable. This indicates that the age-related decline of visual function we report here is likely to be characteristic of the C57Bl/6 strain and not the result of sub-strain differences that can affect the function of the retina.

The optical quality of each eye was scored in two cohorts of aged mice, treated for 8 weeks with systemic elamipretide (SS31) or placebo, by using a modified McDonald–Shadduck scoring scale (Table S1A). The results revealed that no animals in any cohort were found to have fundus abnormalities or cataracts. Three animals in each cohort had corneal opacities that were visible to an observer without the aid of magnification and, under magnification, were scored to be mild (<3); these cases were also categorized as mild, as there was little or no effect on visual acuity (Table S1B and Table S1C). Thus, there was no evidence that the opacities were caused or prevented by elamipretide. It was not feasible to score all cohorts in this detailed manner while maintaining daily treatments and vision testing on multiple cohorts at a time. However, regular eye examinations were performed prior to being tested in the OptoMotry system; when an abnormality (regardless of size or location) was observed in either eye, the animal was removed from the study and not included in any analysis. The number of animals who developed opacities or died during the study are provided in Fig. S1.

## DISCUSSION

We investigated how age affects spatial vision in C57Bl/6 mice, the most widely used mouse strain in neurobiology research. We report that acuity measured under photopic luminance conditions was normal up to ∼18 months of age but declined gradually thereafter until advanced old age (34 months), when <40% normal function remained. Little change in acuity – measured under scotopic luminance conditions – occurred over the same time span. Previous studies have reported that, owing to its affinity for cardiolipin-rich membranes, elamipretide targets mitochondria and has a beneficial effect on mitochondrial function. We, therefore, used elamipretide as a correlative tool to investigate whether mitochondrial dysfunction contributes to age-related visual decline and whether improving mitochondrial function can treat it. We found elamipretide to be effective at treating – i.e. preventing as well as restoring – photopic age-related loss of spatial vision and improving the ability of the visual system to process photopic temporal information.

### Decline of spatial vision with age reflects cone-mediated function

Measures of spatial vision – acuity and contrast sensitivity – report how effectively neurons in the visual system process size and luminance, respectively, in a visual scene. Since changes in spatial visual thresholds reflect changes in the structure and function of the visual system, one of the goals of the study was to identify neural processes that underly age-related visual decline. To this end, we identified a consequential role for cone circuitry in spatial visual decline with age. We report that during more than half of the normal murine lifespan – up to ∼18 months of age–photopic visual function and scotopic spatial visual function was stable. This implies that, in the face of advancing age, the mouse spatial visual system can maintain normal function. The physiology of this resiliency was not investigated in the study. Near 18 months of age, photopic visual function gradually started to decline, with ∼18% deterioration of acuity by 24 months. Proportionally, only little differences in scotopic function were measured up to 26 months.

These results do not imply that photopic changes in spatial vision are the only behavioral changes in the mouse visual system function with age, or that rod-based function is spared the effects of aging. Indeed, there are reports of age-related changes in rod structure and function in mice (Kolesnikov et al., 2010; Rohrer et al., 2003; Wang et al., 2018). There are several possible explanations as to why scotopic spatial vision was not substantially affected by age in our study. One possibility is that the scotopic spatial visual thresholds are normally much lower than photopic thresholds, and as such, it might be more difficult to measure significant changes. Normal adult acuity measured under scotopic conditions (∼0.2 c/d) is ∼50% less than that measured under photopic conditions changes (∼0.39 c/d). Thus, there may be a reduced ability to measure scotopic changes in spatial vision with age. Nevertheless, this explanation seems unlikely, as we measured reductions in scotopic thresholds with age, which were much smaller than the changes in photopic function. This is despite the fact that variability in the measurement of scotopic and photopic measures was similar, and it should have been possible to measure any change if present.

A more likely explanation is that photopic and scotopic vision serves different purposes in mammals, and that spatial vision is more dependent on cone circuitry. Our measurements are unlikely to be a sensitive behavioral test of rod functional decline with age. In humans, rod-driven function is known to decline with age before cone-driven function, which is often manifested as deterioration in peripheral (rod-biased), not foveal (cone-biased) function, and in impaired dark adaptation (Jackson et al., 1999). Thus, the reduced effect of age on scotopic function in our study probably reflects the low-resolution nature of the rod spatial visual system due to the neural pooling of rods, which serves a different function than cone-based circuitry. Mice, although being nocturnal and without a fovea, certainly do not rely on rod-based vision for competent spatial visual function. Instead, they use a higher resolution cone-based system. Thus, our spatial measures, even though they were made using optokinetic responses that do not depend on cortical visual circuits (Douglas et al., 2005), were probably biased towards the detection of functions associated with cone-based circuits, including those that are downstream of the retina. It is possible that different behavioral measures that rely on a different, possibly cortical, functions – such as the Visual Water Task (Prusky et al., 2000) –reveal greater rod-based decline of spatial function with age.

### Restoration of cone-mediated spatial function after treatment with elamipretide

Our study reveals behavioral evidence that elamipretide treatment rather selectively improves age-related loss of photopic visual function. Since elamipretide has been shown to have an affinity for mitochondria and to improve mitochondrial function, our results are also consistent with the possibility that improved mitochondrial function is the mechanism underlying improved visual function. Our results are clinically relevant because age-related visual decline becomes most debilitating in humans when involving cone function linked to spatial vision. We provide a working model of this phenomenon. Indeed, our study supports the prospect that the most debilitating effects of age-related visual decline and disease may be treatable. Elamipretide is in clinical trials to evaluate its ability to ameliorate vision loss in humans [National Library of Medicine (NLM), NCT03891875, NCT02693119] and further studies might be justified to investigate this possibility. In addition to the ability of elamipretide to treat age-related visual decline, it is noteworthy that we found no evidence in young (control) mice that elamipretide treatment leads to better than normal visual function; many therapeutics do not display this feature, which is a common safety concern. Thus, the ability to treat dysfunction without compromising normal function, also lends preclinical support to the safety profile of elamipretide.

The evidence that elamipretide can slow and reverse age-induced decline in visual acuity and contrast sensitivity in aged mice is supported by multiple lines of evidence. This includes the finding that improvements in elamipretide-treated cohorts (relative to placebo) occur over an array of administration start times, treatment durations and types of drug delivery. It also includes the intriguing finding that elamipretide treatment can improve function in mice even at a very advanced age (32 months), albeit less well than in younger animals. We did not investigate the effect elamipretide treatment in relation to age has on temporal neural processing in the visual system as extensively as we did spatial processing. However, the finding that temporal neural processing declines with age (until 26 months) and can be normalized with elamipretide treatment, indicates that the benefits to the visual system of improving mitochondrial function are pervasive. Not only do these convergent results bolster confidence in our conclusion that elamipretide prevents and reverses age-related spatial visual decline, they also indicate that elamipretide is compatible with a wide variety of treatment strategies and ages in humans.

The persistence of the elamipretide treatment benefit after its withdrawal – even following a single dose – is a particularly provocative finding of the study. This indicates that, in addition to elamipretide affecting the function of the visual system while it is biologically active within hours, it can alter the physiology of the visual system in an enduring and beneficial way. This and the finding that the schedule of treatment can affect the rate of recovery, and the rate of decline of the treatment effect, provides both a challenge and an opportunity for translational studies. A challenge because the effects do not appear to follow traditional expectations of pharmacokinetics, an opportunity because elamipretide treatment might be compatible with a wide variety of treatment strategies. Indeed, our data indicate to start an elamipretide treatment regimen with a relatively high ‘loading dose’ to propel improvement of function and to maintain any improvement with a lower dose or more dispersed schedule of treatment. More preclinical studies are necessary to develop a treatment regimen that maximizes the benefit of elamipretide treatment, while minimizing drug exposure.

### Implications regarding future mechanistic studies

This study was neither designed to identify cellular mechanisms of aging, nor to determine how or where mitochondrial function and action are involved in the visual aging process and its remediation. Instead, one of our aims was to generate a detailed description of how age affects spatial visual function. To this end, we presented evidence, derived from >200 mice across several cohorts, that visual aging can be quantified in a way that models the human visual aging process. This is a significant advance because there is now a framework for future studies to test hypotheses regarding the cellular mechanisms of visual aging and to interpret the results in terms of the rate of change over time, not only at single age points. For example, when comparing the spatial vision in young adult mice (i.e. 3 months) to those in mice of advanced age (i.e. 12 months) or even very advanced age (i.e.18 months), our data indicate that no differences would be expected because spatial vision appears to be intact until mice reach the age of 18 months. In addition, our data reveal that comparisons between control groups comprising 18-month-old and older animals, may be more informative than comparisons with young adult (3 months) mice since it would reduce the influence of spurious changes that occur with age, i.e. between 3 and 18 months. Moreover, such studies can now be designed in order to not just test for absolute differences between groups, but also to compare the rate of change over time by using a repeated measures design, for which we have established a baseline here. Such studies promise to have more statistical power to detect small differences than those that make discrete comparisons between two groups.

This study was also designed to test the hypothesis that visual aging is regulated by mitochondrial dysfunction. Our evidence that a drug targeting mitochondria and improving mitochondrial function – i.e. elamipretide – can prevent and restore age-related visual decline is consistent with this hypothesis, and provides an incentive to determine how and where such a process occurs in the visual system. It may seem obvious to investigate whether mitochondrial changes in cones or cone circuits within the retina are the logical place to start. However, since visual behavior is a system level function that involves retina, visual circuits in the brain downstream of the retina and motor circuits that control smooth tracking responses, it is a challenge to link a specific cell type or circuit to a mitochondrial change that is manifest in a visual behavioral change. It is possible that the effects we observed here are solely due to changes in motor function. However, we think this a less likely explanation because the optokinetic tracking task used by us was based on titrating the salience of the visual stimulus, e.g. altering the spatial frequency or contrast, and evaluating the motor response based on any evidence of tracking. We did not use technology to quantify the magnitude of the motor response during optokinetic tracking, which might have revealed a role for a change in the motor response with age and treatment. But even if such study were to reveal a reduction of motor system function with age and improvement upon treatment with elamipretide, it would not eliminate the role for a change in the ability of a visual sensory stimulus to alter optokinetic behavior in an age- and treatment-dependent way.

Exactly which visual circuits are involved in the changes reported are not known, although they are most likely resident in the retina and/or the accessory optic system in the brain (Sun et al., 2015) but not the visual cortex. This is because previous studies have shown that the visual cortex is not part of the circuitry that normally enables optokinetic tracking. Our use of eye drops, in addition to s.c. injections, in this and a previous study (Alam et al., 2015a) represents a limited effort to especially direct the treatment to the eye and retina. That the effects of eye drops were comparable to those of s.c. injections provides encouraging – albeit inconclusive evidence – that the retina is involved in the changes. This is because – when delivered to the surface of the eye made – elamipretide might make its way to the rest of the body via the vascular tissues of the cornea and eyelid.

That we found photopic (cone) function to be selectively altered by age and treatment, however, provides an incentive to determine whether selective changes occur in retinal cones or cone circuit function. Previous reports have provided evidence that elamipretide improves mitochondrial function by interacting with cardiolipin-rich membranes and enhancing formation of respiratory supercomplexes (Birk et al., 2013, 2014, 2015; Szeto, 2014). One strategy to link metabolic change to the effects we have reported here would, therefore, be to measure mitochondrial activity in the mouse retina and retinal pigment epithelium (RPE) RPE-choroid-sclera complex, during aging and in response to elamipretide treatment. Since cones comprise only ∼3% of mouse retinal photoreceptors (Jeon et al., 1998), it would, however, be a challenge to dissociate rod from cone contributions when metabolic changes were observed, or to definitively exclude a role for cones when no changes were found. In addition, that we found changes in temporal neural processing with age and elamipretide treatment in the study, might justify analysis of the flicker fusion electroretinogram (ERG) as an independent way to measure the temporal resolution capabilities of rod and cone photoreceptor systems (Dai et al., 2015). Indeed, when high-frequency visual stimulation is used to produce an ERG, rod responses are saturated by one flash and cannot respond to a subsequent flash; thus, the flicker analysis can be used to detect cone function. If such a flicker fusion study were to confirm a selective ability of the cone system to change with age and elamipretide treatment, not only would it reinforce the findings of this study but it would also link the changes to inner retinal circuits, which are through to enable flicker fusion function (Porciatti and Falsini, 1993).

### Clinical relevance of elamipretide in age-related ocular disease

This study provides evidence that measures of spatial vision based on smooth tracking of moving stimuli can readily identify a treatable form of age-related visual decline. It also provides evidence that age-related decline of spatial visual function can be prevented and partially reversed. Since most measures of spatial visual function in humans do not depend on smooth tracking-based assessments but, instead, rely on ‘stationary stimulus’ measures, such as identifying pictograms or letters on an acuity chart. Thus, it is not clear how our assessments and the effects of a therapy would translate to humans. One way to bridge this gap would be to measure age-related visual decline in humans as well as the effects of a therapy using optokinetic procedures. We have recently introduced such a procedure (Mooney et al., 2018, 2020), which measures spatial vision based on smooth pursuit eye tracking. The application of this methodology to the measurement of age-related visual decline in humans should provide a more-direct way to relate the preclinical findings of this study to human studies. Indeed, the ability to measure contrast sensitivity using this methodology, one might be able to detect age-related visual dysfunction earlier in life – since contrast sensitivity is a more-sensitive measure of spatial visual dysfunction than acuity (Owsley, 2003). The method of drug administration is also of paramount consideration for any new patient intervention. Our results show that eye drops are a viable form of drug administration to treat age-related decline of temporal visual function; the recovery of photopic spatial acuity and contrast sensitivity in response to elamipretide eye drops was comparable to daily s.c. injections of elamipretide.

Finally, our study was not designed to determine whether the effects of elamipretide in the aging visual system are due to the mitochondrial action of the drug. However, the results provide an experimental working hypothesis for future studies to investigate cellular and molecular substrates regarding whether and how age-related mitochondrial dysfunction leads to visual decline, and whether and how improving mitochondrial function enables improvement of visual function. Our results also provide an impetus to investigate whether mitochondrial dysfunction is a treatable pathophysiology of visual aging and age-related visual disease in humans.

## MATERIALS AND METHODS

### Animal subjects

Experiments complied with the policies of Weill Cornell Medicine Animal Care and Use Committee. 224 C57BL/6 mice of either sex, ranging from ∼1-34 months of age, were obtained from National Institute of Aging aged rodent colonies curated by Charles River Laboratories, and from Charles River Laboratories directly, and were group housed at the Burke Neurological Institute vivarium. They had *ad libitum* access to food (Rodent Diet 5053) and acidified water, were maintained at 68-76 F under 30-70% relative humidity, and a photoperiod of 12 h light/dark cycle (06:00 lights on; 18:00 lights off).

### Elamipretide administration

Mice in most experiments were injected subcutaneously (s.c.) once/day with a 1 mg/kg solution of the tetra-peptide elamipretide (also known as SS31), provided by Stealth BioTherapeutics, Newton, MA, USA) dissolved in 0.9% sterile saline (pH 5.5-6.5), or were injected with 0.9% saline alone. In some experiments, mice were administered elamipretide once/day as an ophthalmic-formulated solution (provided by Stealth BioTherapeutics, Newton, MA, USA) daily via eye drops (in 0.01 M sodium acetate buffer solution (pH 6.00); 5 μl/eye), or buffer alone.

### Tests of spatial and temporal visual function

Spatial frequency and contrast thresholds for optokinetic tracking of sine-wave gratings were measured using a virtual optokinetic system (OptoMotry, CerebralMechanics Inc, Medicine Hat, Alberta, Canada; Prusky et al., 2004; Douglas et al., 2005). Vertical sine-wave gratings projected as a virtual cylinder and drifting at 12°/s or gray of the same mean luminance, were displayed on four computer monitors arranged in a square around a small elevated platform. For testing, a mouse was placed on the platform and allowed to move freely. The hub of the cylinder was then centered between the animal's eyes as it shifted its position to maintain the spatial frequency of the grating. Gray was projected when the mouse was ambulating, and a grating was projected when it was stationary, which when visible to the animal, elicited tracking movement of the head and neck. The presence of tracking under each stimulus condition was appraised via live video with a yes/no criterion by an observer blind to the group identity of the mice, and a threshold for tracking was established using a method of limits procedure. A spatial frequency threshold (acuity) – the highest spatial frequency to elicit tracking of a grating at maximal contrast) through each eye (Douglas et al., 2005) – was obtained in a testing session in a few minutes. In some sessions, spatial frequency and contrast thresholds (lowest contrast to elicit tracking) at six spatial frequencies (0.031, 0.064, 0.092, 0.103, 0.192, 0.272 c/d to generate a contrast sensitivity function (CSF)) through each eye separately (by changing the direction of stimulus rotation) were measured (14 thresholds), in ∼30 min. Michelson contrast sensitivity was calculated from the contrast thresholds using the average screen luminance (maximum−minimum)/(maximum+minimum). Experimental animals and their controls were assessed in the same testing session. Thresholds for all animals were obtained under photopic lighting conditions (screen luminance=54 lux), which selectively measures cone-based visual function (Alam et al., 2015b). Photopic measures commenced at one month of age; an age at which mature function is normally established (Prusky et al., 2004). Since it was not feasible to measure the same cohort of mice over their entire life, multiple cohorts that overlapped in age were employed for the study, which enabled the sampling of visual function over the span of adult mouse life. Rod-based function under scotopic conditions (screen luminance =1 lux; Alam et al., 2015b) was assessed in some animals after they were individually dark adapted (>6 h). For this, neutral density (ND) filters (LEE Filters, USA) were placed over the monitor screens (6.9 ND), the testing arena was made light tight, and a near-infrared-sensitive camera with near-infrared lighting (Sony Handycam DCR-HC28, Sony, Japan) was used to image the animals.

To determine whether any spatial visual decline with age, or its remediation with elamipretide, were accompanied by changes in temporal neural processing, we also investigated whether photopic temporal frequency tracking thresholds – a behavioral analog of neural temporal processing (Umino et al., 2008) – was affected by age and elamipretide treatment. For these experiments, the stimulus temporal frequency was maintained at 1.5 Hz; a temporal frequency at which it is feasible to assess tracking responses across a wide range of stimulus speeds. A spatial frequency threshold was then measured in vehicle-treated mice aged 3 or 26 months, and in mice aged 26 months that had been treated daily from the age of 24 months onwards with either s.c. injections of elamipretide (SS31) or vehicle (Placebo).

### Ophthalmic assessments

Optical clarity was evaluated in all test subjects regularly with a visual inspection. A biomicroscope (slit lamp) or dissecting microscope was also used in some cases to inspect the cornea for clarity, size, surface texture and vascularization, and the iris was evaluated for pupil size, constriction, reflected luminescence and synechia. On some occasions, pupils were dilated with a drop of 0.05% tropicamide ophthalmic solution, and the lens was inspected for cataract with an indirect ophthalmoscope or a dissecting microscope (Merriam and Focht, 1962; Worgul et al., 1993). At the same time, the fundus was inspected for damage, degeneration, retinal vessel constriction and optic nerve head abnormalities. The optical quality of the eye was scored in a couple cohorts using a modified McDonald–Shadduck scoring scale (Table S1A) (Hackett and McDonald, 1996). Since it was unfeasible to evaluate optical quality in this way in all cohorts, subjects were simply removed from the study when the ocular health in either eye was found to be compromised during routine visual inspection. Animals with reduced optical clarity or fundus abnormalities were excluded from the analysis; attrition over age is shown in Fig. S1.

### Statistical analyses

Two-way, repeated ANOVA was used to compare groups by using the statistical software package Prism. Post-hoc multiple comparison was performed using Tukey's or Bonferroni's correction methods whenever possible. Statistical comparisons were considered significantly different at *P*<0.05, with many plots showing values of *P*<0.001. In some figures key statistical significances are noted, i.e. *P<0.05, **P<0.01, ***P<0.001. Simple linear or segmental nonlinear regression fit analyses were used to compare data projections in some instances.

## Supplementary Material

Supplementary information
