# Supplementary material for: Treatment of age-related visual impairment with a peptide acting on mitochondria
Source: Dis Model Mech. 2022 Feb 21;15(3):dmm048256. doi: 10.1242/dmm.048256 (PMC8891924; doi:10.1242/dmm.048256)
Supplement: Supplementary information [file dmm-15-048256-s1.pdf]

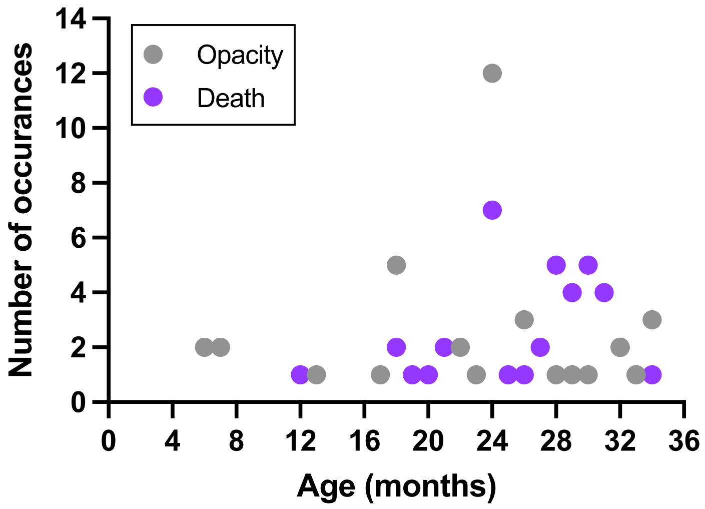

Fig. S1. Study Attrition. Number of recorded opacities and deaths over the course of the study, starting with 412 animals. Animals that unexpectedly died, had to be euthanized due to a health issue or had any gross ocular problem (ie. opacities), were excluded from the study analysis regardless of when the event or problem occurred. Attrition increased with age (deaths, purple; ocular problems, grey). In total, 84 animals were excluded due to opacities and 104 animals due to deaths. 224 animals reached the experimental endpoint with good ocular health.

Table S1A. Ophthalmic Examination – grading of ocular health accompanied by vision measurements. Mice aged 24 or 32 months received daily systemic (subcutaneous) treatment with either Elamipretide (SS31) (red rows) or placebo (blue rows) for 8 weeks. Shown are the raw ophthalmic examination data after 8 weeks of treatment. Both eyes of eachmouse were examined for macroscopic findings and were scored accordingto the Classification System for Grading of Ocular Lesions and OcularPosterior Segment Scoring Scale. Ocular scoring is based on the McDonald–Shadduck Scoring System and was graded depending on effects to ocular tissue and problem severity. The examinations included slit lamp biomicroscopy and fundoscopy. Specifically, the slit lamp examination looked for alterations in the cornea, conjunctiva, iris, anterior chamber, and lens. The retina was examined for gross changes to the retina or optic nerve and noted as normal or abnormal. The pupils were dilated (1.0% tropicamide) to facilitate the fundus examination Grades used were: 0-2, 0-3 and 0-4, where 0 = normal and a higher grader (1-4) = graded abnormality with 4 being the most severe. Treatment was from 24 months (Elamipretide, n=8; placebo, n=10) or 32 months onwards (Elamipretide, n=8; placebo, n=4). In both cohorts we found three animals with small corneal opacities in one eye only. OD, oculus dextrus (right eye); OS, oculus sinister (left eye). Cong (0-3), Conjunctiva Congestion Swell (0-4) , Conjunctiva Swelling Dis (0-3), Conjunctiva Discharge Opac (0-4), Corneal Opacity Area (0-4), Corneal Opacity (% Area) Pann (0-2), Corneal Pannus Aq Flare (0-3), Aqueous Flare Lt Rflx (0-2), Pupillary Light Reflex Iris (0-4), Iris Vessels Lens (N/A), Lens Optic Disc/ Nerve (N/A), N/A = not applicable Ret Blood Ves (N/A), Retinal Blood Vessels Ret Detach (0-3), Retinal Detachment Chor Detach (0-2), Choroid Detachment N/A, normal or abnormal N, normal – grading of ocular health accompanied by vision measurements. Mice aged 24 or 32 months received daily systemic (subcutaneous) treatment with either Elamipretide (SS31) (red rows) or placebo (blue rows) for 8 weeks. Shown are the raw ophthalmic examination data after 8 weeks of treatment. Both eyes of eachmouse were examined for macroscopic findings and were scored accordingto the Classification System for Grading of Ocular Lesions and OcularPosterior Segment Scoring Scale. Ocular scoring is based on the McDonald–Shadduck Scoring System and was graded depending on effects to ocular tissue and problem severity. The examinations included slit lamp biomicroscopy and fundoscopy. Specifically, the slit lamp examination looked for alterations in the cornea, conjunctiva, iris, anterior chamber, and lens. The retina was examined for gross changes to the retina or optic nerve and noted as normal or abnormal. The pupils were dilated (1.0% tropicamide) to facilitate the fundus examination Grades used were: 0-2, 0-3 and 0-4, where 0 = normal and a higher grader (1-4) = graded abnormality with 4 being the most severe. Treatment was from 24 months (Elamipretide, n=8; placebo, n=10) or 32 months onwards (Elamipretide, n=8; placebo, n=4). In both cohorts we found three animals with small corneal opacities in one eye only. OD, oculus dextrus (right eye); OS, oculus sinister (left eye). Cong (0-3), Conjunctiva Congestion Swell (0-4) , Conjunctiva Swelling Dis (0-3), Conjunctiva Discharge Opac (0-4), Corneal Opacity Area (0-4), Corneal Opacity (% Area) Pann (0-2), Corneal Pannus Aq Flare (0-3), Aqueous Flare Lt Rflx (0-2), Pupillary Light Reflex Iris (0-4), Iris Vessels Lens (N/A), Lens Optic Disc/Nerve (N/A), N/A = not applicable Ret Blood Ves (N/A), Retinal Blood Vessels Ret Detach (0-3), Retinal Detachment Chor Detach (0-2), Choroid Detachment N/A, normal or abnormal N, normal

Click here to download Table S1A

Table S1B. Raw vision data analysed using the same cohort as described for Table 1. Weekly spatial frequency thresholds show that - independent of ocular health - Elamipretide-treated animals (red rows) in both cohorts, i.e. treated at either 24 months or at 32 months, improved visual function during the 8-week treatment period compared to placebo-treated animals (blue rows). OD, oculus dextrus (right eye); OS, oculus sinister (left eye).

Click here to download Table S1B

Table S1C. Summary of vision data shown in Tables S1A and B. In each animal, eyesight of both eyes was averaged and standard deviation calculated. These data show little variability between eyes and animals within the same cohort, explaining why error bars are not shown in most panels of Figs 1-3. Stdev, standard deviation. OD, oculus dextrus (right eye); OS, oculus sinister (left eye).

Click here to download Table S1C

Disease Models & Mechanisms • Supplementary information
